# Whole-Genome Sequences of *Vibrio* Species from Warm-Water Shrimps Imported into Canada: Detection of Genetic Elements Associated with Antimicrobial Resistance and Potential Mobilizing Capacities

**DOI:** 10.1128/mra.01014-21

**Published:** 2022-02-03

**Authors:** Januana S. Teixeira, Kelly Weedmark, Forest Dussault, Swapan Banerjee

**Affiliations:** a Bureau of Microbial Hazards, Health Canada, Ottawa, Ontario, Canada; b Bureau of Food Surveillance and Science Integration, Health Canada, Ottawa, Ontario, Canada; Queens College CUNY

## Abstract

We present draft genome sequences of *Vibrio* species (Vibrio alginolyticus, Vibrio cholerae, and two Vibrio parahaemolyticus strains) that were isolated from warm-water shrimps imported into Canada. All four isolates harbor genetic elements associated with antimicrobial resistance (AMR), including mobile genetic elements that can promote horizontal transfer of AMR genes.

## ANNOUNCEMENT

*Vibrio* species are Gram-negative bacteria associated with plankton and estuary-harvested seafood. The genus *Vibrio* includes more than 100 known species, a dozen of which are known to be capable of infecting humans (1). Infections are associated with exposure to seawater or consumption of seafood containing infectious loads of pathogenic biotypes of *Vibrio* species (1, 2). Globally, Vibrio parahaemolyticus is implicated in most seafood-linked foodborne illnesses (3–5). Some Vibrio cholerae strains express cholera toxins, particularly the serovars O1 and O139, which have been associated with multiple pandemics to date and cause life-threatening severe diarrhea (6). Vibrio alginolyticus is an emerging pathogen that is known to be linked to skin infections and mild diarrhea (2).

As part of our surveillance analysis of warm-water shrimps imported into Canada, we isolated and characterized clinically significant *Vibrio* species and identified a subset of strains that exhibited multidrug resistance (MDR), defined as resistance to three or more antibiotics. Here, we report the whole-genome sequences (WGS) of four MDR *Vibrio* strains. These strains were isolated and characterized at the species level as described previously (7) and were stored frozen at −80°C, with antimicrobial resistance (AMR) profiles determined by the Kirby-Bauer disk diffusion method (8, 9). The antibiotic susceptibility test results showed resistance to up to nine different antibiotics (Table 1). For DNA isolation, stock cultures were struck onto tryptic soy agar with 2% NaCl (TSA-2N) (Difco BD, Franklin Lakes, NJ, USA), and single colonies were grown overnight at 35°C on TSA-2N. Genomic DNA was extracted using the Maxwell 16-cell DNA purification kit (Promega, Madison, WI, USA), and indexed libraries were prepared using the Nextera XT kit and sequenced on a MiSeq instrument (2 × 300-bp paired-end reads, v3 chemistry) according to the manufacturer’s protocol (Illumina, San Diego, CA, USA). Sequence analysis tools were used with default settings for read adapter trimming, quality filtering, and error correction (BBMap v38.26 [BBDuk and Tadpole]) (https://sourceforge.net/projects/bbmap), *de novo* assembly (SKESA v2.3 [SVN 551987:557549M] and Pilon v1.22) (10), gene prediction (Prodigal [commit fe80417]) (11), and summary metrics (QUAST v5.0.0 [de6973bb]) (12). AMR gene and plasmid characterizations were predicted by in-house scripts using the Resistance Gene Identifier (RGI) v5.0.0/Comprehensive Antibiotic Resistance Database (CARD) v3.0.3 (13) and MOB-suite v1.4.9 tools and database (14). Isolate details are summarized in Table 1.

**TABLE 1:** Genome profiles and AMR results for the four *Vibrio* strains

Strain no.	Organism	GenBank accession no.	SRA accession no.	Genome size (Mbp)	No. of reads	Genome coverage (×)	No. of contigs	*N*_50_ (bp)	GC content (%)	No. of genes	AMR phenotype^*a*^	Plasmid type	Predicted mobility	Plasmid genotype
ISF-22-6	V. alginolyticus	JAILXO010000000	SRR15526828	5.2	468,108	90	73	343,022	44.4	4,719	AMP, SXT, TE, SF, OT (E, KF)	Unknown	Nonmobilizable	*CARB-19*, *tet*(35), *aph(3″)-Ib*, *aph(6)-Id*, *floR*, *sul2*
ISF-208-6	V. cholerae	JAILXN010000000	SRR15526827	4.4	726,913	160	82	206,565	47.4	4,127	AMP, CTX, K, S, KF, CTF, PIP, OX, E (ENO)	IncA/C_2_	Conjugative	*varG*, *catB9*, *sul1*, *aac(6′)-IId*, *catB3*, *sul1*, *mphA*, *PER3*
ISF-232-3	V. parahaemolyticus	JAILXM010000000	SRR15526826	5.1	190,219	30	161	80,238	45.3	4,639	AMP, CTX, K, KF, CTF (E)	IncQ1	Mobilizable	*aac(6′)-IIa*, *ANT(2″)-Ia*, *VEB-5*
ISF-238-3	V. parahaemolyticus	JAILXL010000000	SRR15526825	5.4	592,288	465	85	195,494	45.3	4,990	AMP, S, SF, OT (CTF, E, KF)	Unknown	Nonmobilizable	*aph(3″)-Ib*, *aph(6)-Id*, *floR*, *sul2*

a AMP, ampicillin; CTF, ceftiofur; CTX, cefotaxime; E, erythromycin; ENO, enrofloxacin; K, kanamycin; KF, cephalothin; OT, oxytetracycline; OX, oxolinic acid; PIP, piperacillin; S, streptomycin; SF, sulfafurazole; SXT, sulfamethoxazole-trimethoprim; TE, tetracycline. Abbreviations shown in parentheses refer to intermediate resistance.

A V. parahaemolyticus strain isolated in-house from Canadian molluscan shellfish was shown to harbor an integrative and conjugative element (ICE) belonging to the SXT/R391 family, located on chromosome I (15). This type of mobilizing capacity is known to be associated with adaptation and evolution (16), including acquired AMR traits. The widespread detection of several vector systems in Gram-negative bacteria has been reported and reviewed (17). WGS of the submitted *Vibrio* species with diverse extrachromosomal elements are available for the assessment of various *in silico* tools to predict phenotypic AMR detected in the *Vibrio* isolates using standard laboratory procedures (8, 9) and for the advancement and improvement of WGS-based prediction of AMR phenotypes.

### Data availability.

The WGS data have been deposited in EMBL/GenBank as BioProject PRJNA645603, under the accession numbers listed in Table 1.

## References

[1] Morris JG, Jr, Acheson D. 2003. Cholera and other types of vibriosis: a story of human pandemics and oyster on the half shell. Clin Infect Dis 37:272–280. doi:10.1086/375600. 12856219

[2] Dechet AM, Yu PA, Koram N, Painter J. 2008. Nonfoodborne *Vibrio* infections: an important cause of morbidity and mortality in the United States, 1997–2006. Clin Infect Dis 46:970–976. doi:10.1086/529148. 18444811

[3] DePaola A, Nordstrom JL, Bowers JC, Wells JG, Cook DW. 2003. Seasonal abundance of total and pathogenic *Vibrio parahaemolyticus* in Alabama oysters. Appl Environ Microbiol 69:1521–1526. doi:10.1128/AEM.69.3.1521-1526.2003. 12620838 PMC150055

[4] Ceccarelli D, Hasan NA, Huq A, Colwell RR. 2013. Distribution and dynamics of epidemic and pandemic *Vibrio parahaemolyticus* virulence factors. Front Cell Infect Microbiol 3:97. doi:10.3389/fcimb.2013.00097. 24377090 PMC3858888

[5] Banerjee SK, Kearney AK, Nadon CA, Peterson CL, Tyler K, Bakouche L, Clark CG, Hoang L, Gilmour MW, Farber JM. 2014. Phenotypic and genotypic characterization of Canadian clinical isolates of *Vibrio parahaemolyticus* collected from 2000 to 2009. J Clin Microbiol 52:1081–1088. doi:10.1128/JCM.03047-13. 24452166 PMC3993483

[6] World Health Organization. 2009. Cholera: global surveillance summary, 2008. Wkly Epidemiol Rec 84:309–324. 19645127

[7] Banerjee SK, Farber JM. 2017. Detection, enumeration and isolation of *Vibrio parahaemolyticus* and *V. vulnificus* from seafood: development of a multidisciplinary protocol. J AOAC Int 100:445–453. doi:10.5740/jaoacint.16-0290. 28118133

[8] Bauer AW, Kirby WMM, Sherris JC, Turck M. 1966. Antibiotic susceptibility testing by a standardized single disk method. Am J Clin Pathol 45:493–496. doi:10.1093/ajcp/45.4_ts.493. 5325707

[9] Clinical and Laboratory Standards Institute. 2015. Methods for antimicrobial dilution and disk susceptibility testing of infrequently isolated or fastidious bacteria, 3rd ed. CLSI M45. Clinical and Laboratory Standards Institute, Wayne, PA. 10.1086/510431 17173232

[10] Souvorov A, Agarwala R, Lipman D. 2018. SKESA: strategic k-mer extension for scrupulous assemblies. Genome Biol 19:153. doi:10.1186/s13059-018-1540-z. 30286803 PMC6172800

[11] Hyatt D, Chen GL, Locascio PF, Land ML, Larimer FW, Hauser LJ. 2010. Prodigal: prokaryotic gene recognition and translation initiation site identification. BMC Bioinformatics 11:119. doi:10.1186/1471-2105-11-119. 20211023 PMC2848648

[12] Gurevich A, Saveliev V, Vyahhi N, Tesler G. 2013. QUAST: quality assessment tool for genome assemblies. Bioinformatics 29:1072–1075. doi:10.1093/bioinformatics/btt086. 23422339 PMC3624806

[13] Jia B, Raphenya AR, Alcock B, Waglechner N, Guo P, Tsang KK, Lago BA, Dave BM, Pereira S, Sharma AN, Doshi S, Courtot M, Lo R, Williams LE, Frye JG, Elsayegh T, Sardar D, Westman EL, Pawlowski AC, Johnson TA, Brinkman FSL, Wright GD, McArthur AG. 2017. CARD 2017: expansion and model-centric curation of the comprehensive antibiotic resistance database. Nucleic Acids Res 45:D566–D573. doi:10.1093/nar/gkw1004. 27789705 PMC5210516

[14] Robertson J, Nash JHE. 2018. MOB-suite: software tools for clustering, reconstruction and typing of plasmids from draft assemblies. Microb Genomics 4:e000206. doi:10.1099/mgen.0.000206. PMC6159552 30052170

[15] Bioteau A, Huguet K, Burrus V, Banerjee S. 2018. Genome sequence of a Canadian *Vibrio parahaemolyticus* isolate with unique mobilizing capacity. Genome Announc 6:e00520–18. doi:10.1128/genomeA.00520-18. 29903822 PMC6003736

[16] Burrus V, Waldor MK. 2004. Shaping bacterial genomes with integrative and conjugative elements. Res Microbiol 155:376–386. doi:10.1016/j.resmic.2004.01.012. 15207870

[17] Hazen TH, Pan L, Gu JD, Sobecky PA. 2010. The contribution of mobile genetic elements to the evolution and ecology of *Vibrios*. FEMS Microbiol Ecol 74:485–499. doi:10.1111/j.1574-6941.2010.00937.x. 20662928

